# Increased Reactive Oxygen Species Generation Contributes to the Atherogenic Activity of the B2 Bradykinin Receptor

**DOI:** 10.3389/fmed.2019.00032

**Published:** 2019-02-21

**Authors:** Alexander Perhal, Stefan Wolf, Yahya F. Jamous, Andreas Langer, Joshua Abd Alla, Ursula Quitterer

**Affiliations:** ^1^Molecular Pharmacology, Department of Chemistry and Applied Biosciences, ETH Zurich, Zurich, Switzerland; ^2^Department of Medicine, Institute of Pharmacology and Toxicology, University of Zurich, Zurich, Switzerland

**Keywords:** BDKRB2, reactive oxygen species, atherosclerosis, hypercholesterolemia, GCH1, Apoe, AGTR1, ACE

## Abstract

Atherosclerosis and ensuing cardiovascular disease are major causes of death with insufficient treatment options. In search for pathomechanisms of atherosclerosis, we investigated the impact of the B2 bradykinin receptor, *Bdkrb2*, on atherosclerotic lesion formation, because to date it is not clear whether the B2 bradykinin receptor is atheroprotective or atherogenic. As a model of atherosclerosis, we used hypercholesterolemic *ApoE*-deficient (apolipoprotein E-deficient) mice, which develop atherosclerotic lesions in the aorta with increasing age. The role of *Bdkrb2* in atherosclerosis was studied in *ApoE*-deficient mice, which were either *Bdkrb2*-deficient, or had moderately increased aortic B2 bradykinin receptor protein levels induced by transgenic *BDKRB2* expression under control of the ubiquitous CMV promoter. We found that *Bdkrb2* deficiency led to a significantly decreased atherosclerotic plaque area whereas transgenic *BDKRB2* expression enhanced atherosclerotic lesion formation in the aorta of *ApoE*-deficient mice at an age of 8 months. Concomitantly, the aortic content of reactive oxygen species (ROS) was higher in *BDKRB2*-expressing mice whereas *Bdkrb2* deficiency decreased aortic ROS levels of *ApoE*-deficient mice. In addition, aortic nitrate as a marker of nitric oxide activity and the endothelial nitric oxide synthase (eNOS) co-factor, tetrahydrobiopterin (BH4) were reduced in *BDKRB2*-expressing *ApoE*-deficient mice. The decreased aortic BH4 content could be a consequence of increased ROS generation and down-regulated aortic expression of the BH4-synthesizing enzyme, *Gch1* (GTP cyclohydrolase 1). In agreement with a causal involvement of decreased BH4 levels in the atherogenic function of *BDKRB2*, we found that treatment with the BH4 analog, sapropterin, significantly retarded atherosclerotic plaque formation in *BDKRB2*-expressing *ApoE*-deficient mice. Together our data show that the B2 bradykinin receptor is atherogenic, and the atherosclerosis-promoting function of *BDKRB2* is partially caused by decreased aortic BH4 levels, which could account for eNOS uncoupling and further enhancement of ROS generation.

## Introduction

Atherosclerosis and cardiovascular disease are leading causes of death worldwide (1, 2). The high morbidity and mortality of atherosclerotic vascular disease is in part attributed to limited treatment options (1, 2). Elucidation of pathomechanisms with the identification of potential new targets to improve the pharmacotherapy of atherosclerosis therefore is of great interest (1–3). To study pathomechanisms of atherosclerosis, hypercholesterolemic, apolipoprotein E (*ApoE*)-deficient mice are often used as a model because these mice reproduce major features of atherosclerotic vascular disease such as hypercholesterolemia-induced atherosclerotic lesion formation in the vascular system with increasing age (4–8).

In this study, we investigated the role of the B2 bradykinin receptor (*BDKRB2*) in atherosclerotic lesion formation. To date it is not known whether *BDKRB2* is atheroprotective or atherogenic. Due to the blood pressure-lowering and nitric oxide- (NO)-generating activity, the B2 bradykinin receptor is considered to exert cardioprotection (9, 10). This cardioprotective potential of B2 bradykinin receptor stimulation by the agonist bradykinin and related kinins of the kinin-kallikrein system is exploited therapeutically with ACE (angiotensin-converting enzyme) inhibitors. ACE inhibition not only blunts the generation of the vasopressor angiotensin II but also prevents the proteolytic degradation of bradykinin (9, 10). The contribution of bradykinin to the antihypertensive activity of ACE inhibitors is well-documented in experimental models and patients (11, 12). On the other hand, the atherosclerosis-decreasing potential of ACE-inhibition is reportedly independent from bradykinin and B2 bradykinin receptor stimulation (13). Moreover, bradykinin and related kinins are pro-inflammatory peptides, and inflammation is an established risk factor of atherosclerosis (14, 15). In addition, beneficial B2 bradykinin receptor-stimulated nitric oxide (NO) generation and vasodilation are impaired in atherosclerosis (16). Hypercholesterolemia and atherosclerosis are known to cause endothelial dysfunction with concomitant uncoupling of endothelial nitric oxide synthase (eNOS), which then generates atherosclerosis-promoting reactive oxygen species (ROS) instead of atheroprotective NO (17–19).

In view of this scenario, we investigated the impact of the B2 bradykinin receptor on atherosclerotic lesion development. To study the role of the B2 bradykinin receptor (*Bdkrb2*) in atherosclerosis, we used (i) *ApoE*–/– mice with endogenously expressed *Bdkrb2*, (ii) *ApoE*–/– mice with *Bdkrb2* deficiency, and (iii) *ApoE*–/– mice with moderately increased transgenic *BDKRB2* expression level. We found that transgenic B2 receptor expression enhanced atherosclerotic plaque formation in the aorta of *ApoE*–/– mice whereas *Bdkrb2* deficiency retarded the development of atherosclerosis.

## Materials and Methods

### Experimental Model of Atherosclerosis, and Generation of Transgenic Mice

The study was performed with three groups of male mice, i.e., (i) apolipoprotein E-deficient (*ApoE*–/–) mice in B6 (C57Bl/6J) background (4–8), (ii) double-deficient *Bdkrb2*–/–*ApoE*–/– mice (B2–/–*ApoE*–/–), and (iii) *ApoE*–/– mice with transgenic expression of *BDKRB2* under control of the ubiquitous CMV immediate-early promoter/enhancer (derived from plasmid pcDNA3, Invitrogen AG - Thermo Fisher Scientific). *Bdkrb2*-deficient mice in B6 background were obtained by backcross breeding of *Bdkrb2*–/– mice (20) for ten generations into B6 background. Double-deficient, *Bdkrb2*–/– and *ApoE*–/– mice, were subsequently generated by cross-breeding, and identified by genotyping PCR. For transgenic expression of *BDKRB2*, the *BDKRB2* transgene (2 ng/μL) was injected into the pronucleus of fertilized oocytes isolated from super-ovulated *ApoE*–/– mice with B6 background followed by transfer of 2-cell embryos into pseudo-pregnant CD-1 foster mice similarly as described (21). After weaning at an age of 3–4 weeks, PCR genotyping was performed with ear-punch biopsies. Founder mice of the FO generation were identified with stable integration of the transgenic DNA into the genomic mouse DNA and used for further breeding. Due to the pathologic phenotype in *ApoE*–/– background, Tg-CMVBDKRB2 (Tg-B2++) mice with *ApoE*-deficiency only were used for phenotyping but otherwise, the colony of Tg-CMVBDKRB2 mice was maintained on the B6 background without *ApoE*-deficiency (C57BL/6-Tg-(CMVBDKRB2)Sjaa; Janvier No. 181.281 ETH Zurich). Phenotyping was determined with 8-month-old mice. Mice were kept on a 12 h light/12 h dark cycle, had free access to food and water, and were fed a standard rodent chow diet (Ain-93-based diet without addition of tocopherol acetate) containing 7% fat and 0.15% cholesterol. As indicated, 3-month-old mice received sapropterin (10 mg/kg/d, in drinking water, freshly prepared, every day) for 5 months. Treatment of Tg-B2++*ApoE*–/– mice and *ApoE*–/– mice with the ACE inhibitor, captopril (20 mg/kg/d, in drinking water, freshly prepared, every day) was started at an age of 3 months and continued for 5 months until the end of the observation period at 8 months. At the end of the study, anesthetized mice (ketamine/xylazine 100 mg/10 mg per kg body-weight) were perfused intracardially with ice-cold, sterile PBS, the aorta was rapidly dissected on ice and processed for further analysis. All animal experiments were performed according to NIH guidelines, and reviewed and approved by the local committee on animal care and use (Cantonal Veterinary office, Zurich).

### Biochemical Assays and Aortic Atherosclerotic Lesion Determination

The atherosclerotic lesion area was determined in the aorta by quantitative image analysis of oil red O-stained aortas opened longitudinally (7, 8). The area of the aortic intima with pathologic intimal atherosclerotic lesions was determined with hematoxylin-eosin-stained, aortic paraffin sections of the aortic arch. The aortic content of BH4 was determined as described (22). The content of reactive oxygen species (ROS) was determined by quantitative fluorescence evaluation of dihydroethidium- (DHE)-stained aortic cryosections (8, 23). The aortic expression of *Gch1* (GTP cyclohydrolase 1) was assessed after reverse transcription of mRNA into cDNA followed by quantitative real-time qRT-PCR using a LightCycler 480 Instrument (Roche). For quantitative real-time qRT-PCR, total aortic RNA was isolated by the RNeasy Mini kit according to the protocol of the manufacturer (Qiagen). RNA purity was confirmed by an absorbance ratio A260/280 of ~2.0. The absence of RNA degradation and RNA quality were further controlled by the presence of bright bands of 18S and 28S ribosomal RNA in denaturing RNA electrophoresis. RNA was reverse transcribed into cDNA by the Transcriptor High Fidelity cDNA Synthesis Kit and subjected to qRT-PCR using the LightCycler® 480 System with the LightCycler® 480 SYBR Green I Master reaction mix according to the protocol of the manufacturer (Roche Molecular Systems). Primer sequences used for determination of *Gch1* expression by qRT-PCR were as follows: Gch1 forward 5′-GCCGCTTACTCGTCCATTCT-3′, and Gch1 reverse 5-CCACCGCAATCTGTTTGGTG-3′. Specific amplification of the *Gch1* fragment of 358 bp was controlled by agarose gel electrophoresis. Total number of B2 bradykinin receptor binding sites was determined with aortic smooth muscle cells in HEPES-buffered DMEM (supplemented with 1% BSA, protease inhibitors and enalaprilat) by saturation radioligand binding (for 2 h at 4°C) with increasing concentrations (0.1–10 nM) of [2,3-prolyl-3,4-^3^H(N)]bradykinin (79-96 Ci/mmol; Perkin Elmer) in the absence and presence of 10 μM HOE140 to determine non-specific binding. Likewise, the number AT1 receptor binding sites was determined with Sar^1^,[^125^I]Tyr^4^,Ile^8^-angiotensin II (2200 Ci/mmol; Perkin Elmer) in the absence and presence of 10 μM losartan. Aortic vascular smooth muscle cells were isolated from aortas of *ApoE*–/–, *Bdkrb2*–/–*ApoE*–/–, Tg-B2++*ApoE*–/– and non-transgenic B6 control mice at an age of 6–10 days. For vascular smooth muscle cell (VSMC) isolation, aortas from 10 to 15 mice were dissected. Aortas were endothelium-denuded and minced with a scalpel followed by a washing step with HEPES-buffered DMEM (supplemented with 1 mM L-glutamine, 100 I.U./ml penicillin and 100 μg/ml streptomycin) and digestion at 37°C with collagenase (2 mg/ml of collagenase type 2) under constant agitation with a magnetic stirrer. The first digestion step was discarded and in subsequent steps, the enzyme solution was exchanged every 5 min. Cells were filtered through a nylon mesh (pore size 40 μm) and collected in DMEM supplemented with 10% FCS. After the aortic tissue was digested, cell fractions were pooled. Thereafter, cells were collected by centrifugation (300 x g), plated on cell culture plates and cultured in DMEM supplemented with 10% FCS as described (24). Total inositol phosphates of aortic smooth muscle cells were determined as described (25). The specific B2 bradykinin receptor-stimulated signal is given and was determined by bradykinin stimulation (100 nM) in the absence and presence of 10 μM of the B2-specific antagonist, HOE140. Aortic Gch1 protein contents were determined by immunoblot detection with GCH1/Gch1-specific antibody (GCH1 monoclonal antibody M01, clone 4A12, Cat. No. H00002643-M01, Abnova) similarly as detailed previously (25). Aortic tissue nitrate content was determined with a fluorometric assay kit according to the protocol of the manufacturer (Cayman Chemical).

### Statistical Analysis

All data are presented as mean ± s.d. Analysis of variance followed by a Post-test as indicated was performed to determine statistical significance between more than two groups. Statistical significance was set at a *p*-value of < 0.05. Statistical evaluation was performed with GraphPad PRISM 7.0.

## Results

### Increased Number of Aortic B2 Bradykinin Receptors in Tg-B2++*ApoE–/–* Mice

**Figure 1:**
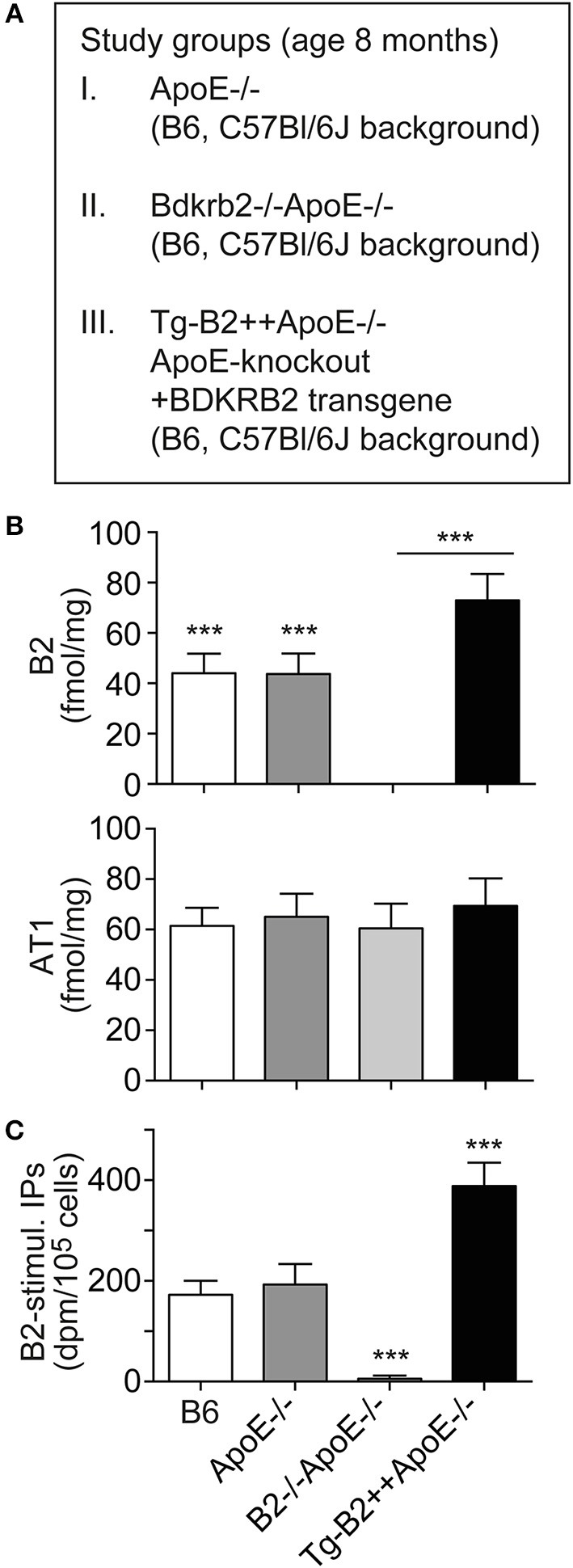
Increased number of aortic B2 bradykinin receptors in Tg-B2++*ApoE*–/– mice. **(A)** Overview of three study groups of *ApoE*–/– mice. **(B)** B2 bradykinin receptor binding sites (upper) and angiotensin II AT1 receptor binding sites (lower) were determined with aortic vascular smooth muscle cells isolated from three study groups of *ApoE*–/– mice and non-transgenic B6 mice (mean ± s.d.; *n* = 6; ^***^p < 0.001 (B6 and *ApoE*–/– vs. B2–/–*ApoE*–/– and Tg-B2++*ApoE*–/–); Tukey‘s test). **(C)** The specific B2 bradykinin receptor-stimulated increase in total inositol phosphate levels (B2-stimul. IPs) was determined with aortic vascular smooth muscle cells isolated from different study groups (±s.d.; *n* = 3 biological replicates; ^***^p < 0.001 (Tg-B2++*ApoE*–/–) vs. B6 and *ApoE*–/–; ^***^p < 0.001 (B2–/–*ApoE*–/–) vs. B6, *ApoE*–/–, and Tg-B2++*ApoE*–/–; Tukey‘s test).

To investigate the role of the B2 bradykinin receptor in atherosclerosis, we used hypercholesterolemic *ApoE*–/– mice as an experimental model of atherosclerosis. These mice develop atherosclerotic lesions in the vascular system and the aorta with increasing age (4–8). In frame of our study, we compared three groups of *ApoE*–/– mice with different expression levels of the B2 bradykinin receptor, i.e., (i) *ApoE*–/– mice in B6 (C57Bl/6J) background with endogenous *Bdkrb2* expression level, (ii) *ApoE*–/– mice in B6 background, which are deficient in the B2 bradykinin receptor gene, *Bdkrb2–/–ApoE-/* (B2–/–*ApoE*–/–), and (iii) *ApoE*–/– mice in B6 background with transgenic expression of *BDKRB2* under control of the ubiquitous CMV promoter, Tg-B2++*ApoE*–/– mice (Figure 1A). Because the genetic background has a strong influence on the atherosclerotic phenotype of *ApoE*–/– mice (26), all groups of transgenic mice used in our study had identical B6 background. To obtain *Bdkrb2*–/– mice with pure B6 (C57Bl/6J) background, we performed 2 years of backcrossing of *Bdkrb2*–/– mice into the B6 background for more than 10 generations. These *Bdkrb2*–/– mice with B6 background were then used for cross-breeding with *ApoE*–/– mice, to finally obtain homozygous mice, which were double-deficient in *Bdkrb2* and *ApoE* (*Bdkrb2*–/– and *ApoE*–/–). To generate mice with moderately increased *BDKRB2* level, we generated *BDKRB2*-transgenic mice in *ApoE*–/– background with expression of *BDKRB2* under control of the ubiquitous CMV promoter. We used the CMV promoter, because the endogenous B2 bradykinin receptor is also ubiquitously expressed. Male offspring of these three study groups of mice with different expression levels of the B2 bradykinin receptor were used to analyze the impact of this receptor on the pathogenesis of atherosclerosis (Figure 1A).

We initially characterized the vascular B2 bradykinin receptor content of study groups. B2 bradykinin receptor levels on aortic vascular smooth muscle cells (VSMC) isolated from different transgenic mouse lines were determined by radioligand binding. Our data show that B2 bradykinin receptor levels were moderately increased on VSMC of Tg-B2++*ApoE*–/– mice compared to *ApoE*–/– mice, i.e., the total number of B2 receptor bindings sites was 72.9 ± 10.5 fmol/mg on Tg-B2++*ApoE*–/– VSMC compared to 43.7 ± 8.2 fmol/mg in *ApoE*–/– mice with endogenous *Bdkrb2* level and 44.1 ± 7.7 fmol/mg in non-transgenic B6 mice (Figure 1B). As a control, the B2 bradykinin receptor was absent in VSMC from double-deficient, B2–/–*ApoE–/–* mice (Figure 1B). In contrast to the B2 bradykinin receptor, numbers of angiotensin II AT1 receptor binding sites were not significantly different between all study groups (Figure 1B). Endogenously expressed and transgenic B2 bradykinin receptors of aortic smooth muscle cells were functional and mediated an increase in total inositol phosphate levels upon bradykinin stimulation (Figure 1C).

Together these data show that transgenic *BDKRB2* expression under control of the ubiquitous CMV promoter led to a moderately increased number of B2 bradykinin receptor binding sites on aortic smooth muscle cells of Tg-B2++*ApoE*–/– mice whereas the B2 receptor was absent in B2–/–ApoE–/– mice with deficiency of the B2 bradykinin receptor gene, *Bdkrb2*.

### Transgenic *BDKRB2* Expression Enhances Atherosclerotic Lesion Formation Whereas Bdkrb2 Deficiency Leads to a Decreased Atherosclerotic Plaque Area in the Aorta of *ApoE–/–* Mice

**Figure 2:**
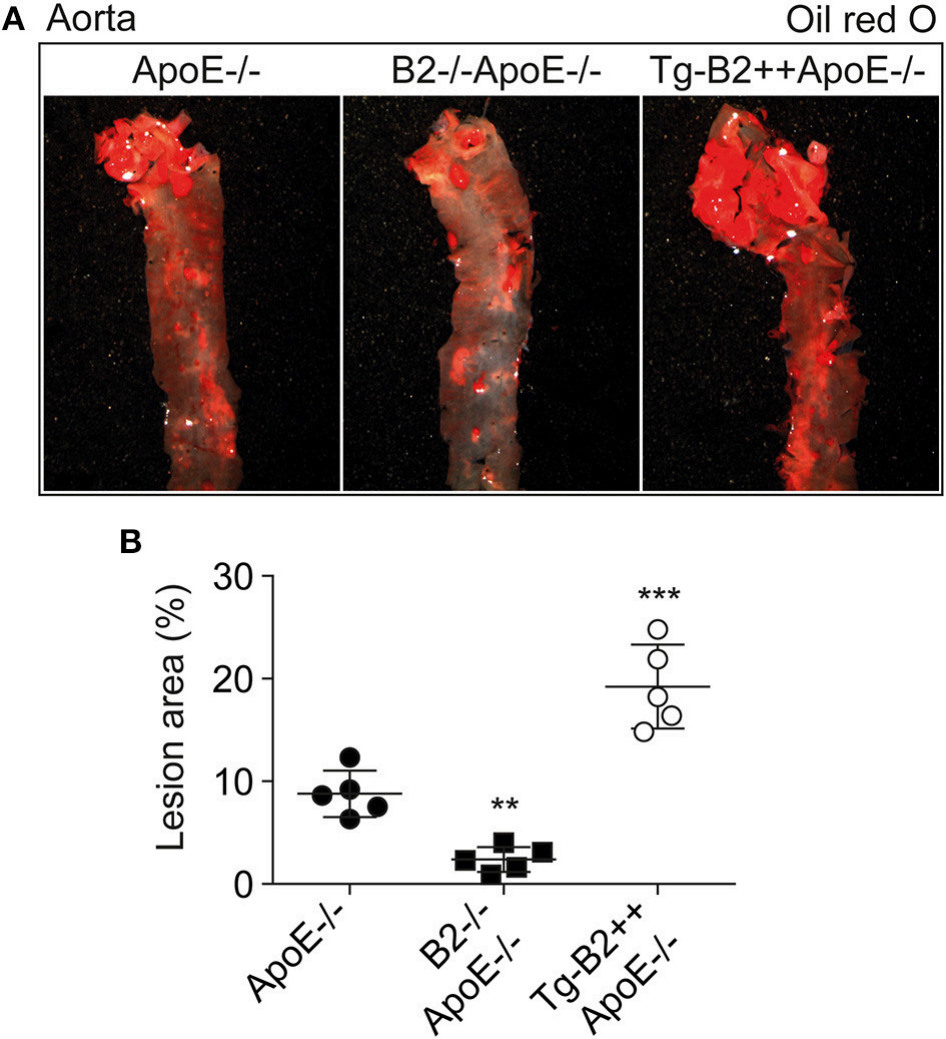
Transgenic *BDKRB2* expression enhances atherosclerotic lesion formation whereas *Bdkrb2* deficiency leads to a decreased atherosclerotic plaque area in the aorta of *ApoE*–/– mice. **(A,B)** Atherosclerotic lesion area was determined with oil red O-stained aortas by quantitative image analysis. Representative aortic images are shown in **(A)**, and **(B)** shows quantitative data evaluation (mean ± s.d.; *n* = 5; ^**^p < 0.01 and ^***^p < 0.001 vs. *ApoE*–/–; Dunnett‘s test).

To investigate the impact of the B2 bradykinin receptor on atheroma formation, we determined the atherosclerotic lesion area in the aorta of 8-month-old *ApoE*–/– mice. As outlined before, the study compared three different groups of mice, i.e., (i) *ApoE*–/– mice with endogenous *Bdkrb2* expression level, (ii) B2–/–A*poE*–/– mice with *Bdrkb2* deficiency, and (iii) Tg-B2++*ApoE*–/– mice with moderately increased B2 receptor level due to transgenic *BDKRB2* expression. All mice had B6 background. Atherosclerotic lesion area in the aorta was quantified by oil red O staining (Figure 2A). We found that transgenic *BDKRB2* expression led to a significantly enhanced atherosclerotic plaque formation in the aorta of Tg-B2++*ApoE*–/– mice, i.e., the atherosclerotic lesion area was increased 2.2 ± 0.4-fold in Tg-B2++*ApoE*–/– mice compared to *ApoE*–/– mice with endogenous *Bdkrb2* level (Figure 2B). In contrast, B2 bradykinin receptor deficiency led to a significantly decreased atherosclerotic plaque area in double-deficient, *B2*–/–*ApoE*–/– mice compared to ApoE–/– mice with endogenously expressed *Bdkrb2* (Figure 2B). These data provide strong evidence that the B2 bradykinin receptor enhances the progression of atherosclerotic plaque formation in the aorta of 8-month-old *ApoE*–/– mice as an experimental model of atherosclerosis.

### Transgenic *BDKRB2* Expression Leads to an Increased Aortic ROS Content in Tg-B2++*ApoE*–/– Mice

**Figure 3:**
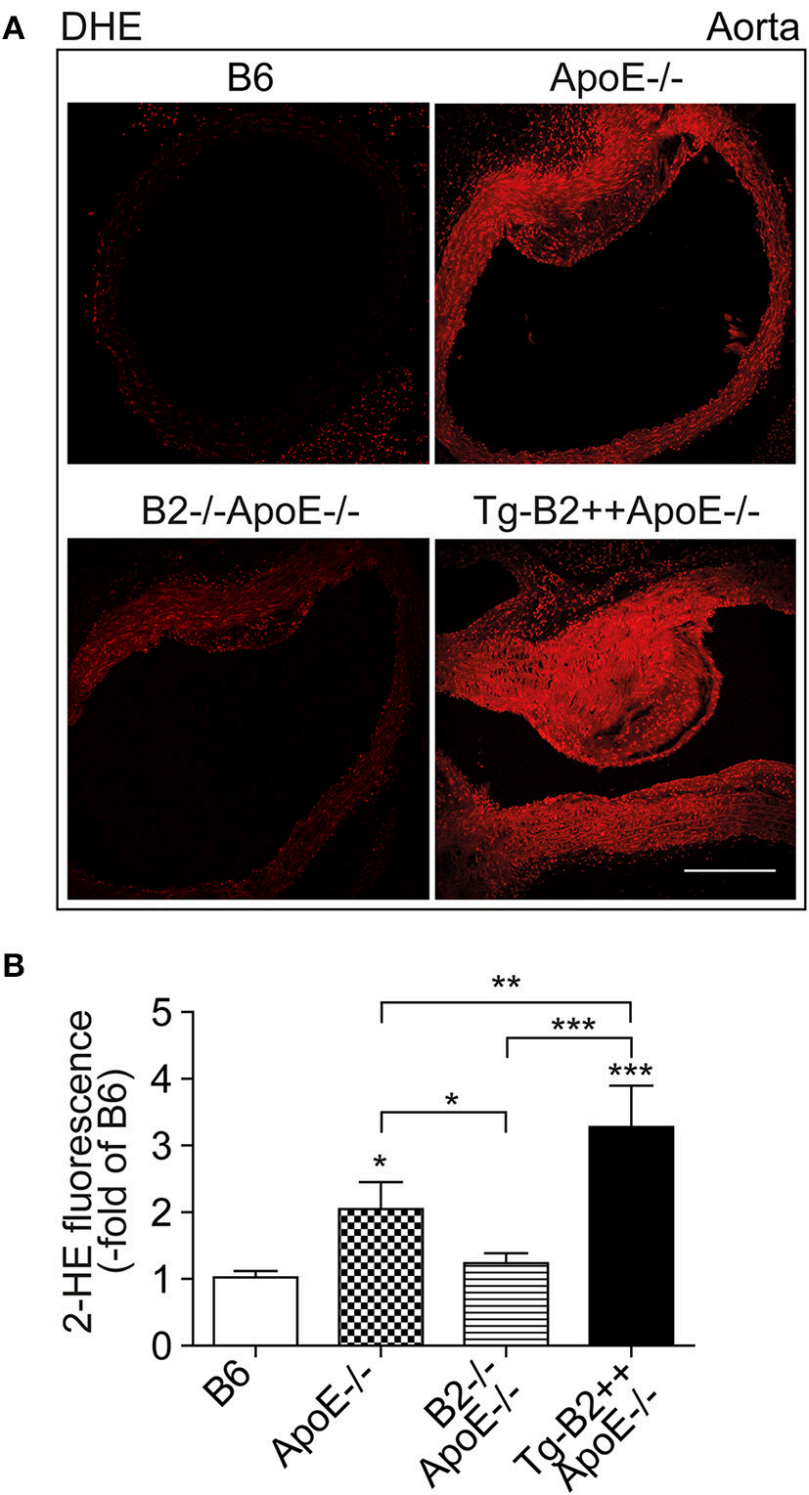
Transgenic BDKRB2 expression leads to an increased aortic ROS content in Tg-B2++ApoE–/– mice. **(A,B)** The aortic ROS content was determined by DHE staining of aortas from different study groups. Representative images are shown in **(A)**, and **(B)** shows quantitative data (mean ± s.d.; *n* = 4; ^*^p < 0.05 (ApoE–/–) and ^***^p < 0.001 (Tg-B2++ApoE–/–) vs. B6; ^**^p < 0.01; other comparisons are indicated; Tukey‘s test; bar: 100 μm).

Enhanced generation of reactive oxygen species (ROS) is a major contributor to atherosclerotic plaque formation in experimental models of atherosclerosis and involved in the pathogenesis of atherosclerosis in patients (8, 18, 27). We asked whether the B2 bradykinin receptor led to an increased aortic ROS content of Tg-B2++*ApoE*–/– mice. Aortic ROS was detected *in situ* by dihydroethidium (DHE) staining (8, 23). Quantitative image analysis shows that the ROS production in the aorta of *BDKRB2*-expressing Tg-B2++*ApoE*–/– mice was significantly increased compared to *ApoE*–/– mice with endogenous B2 receptor expression level, i.e., aortic ROS levels of Tg-B2++*ApoE*–/– mice were 1.6 ± 0.3-fold higher than those of *ApoE*–/– mice (Figures 3A,B). Vice versa, there was a significant decrease in the aortic ROS content of double-deficient *B2*–/–*ApoE*–/– mice compared to *ApoE*–/– mice with intact *Bdkrb2* gene (Figures 3A,B). Taken together, the B2 bradykinin receptor mediates an enhanced production of ROS in the aorta of atherosclerosis-prone Tg-B2++*ApoE*–/– mice with hypercholesterolemia.

### The Aortic Nitrate Content as a Marker of NO Activity and the Nitric Oxide Synthase (NOS) Cofactor Tetrahydrobiopterin (BH4) Are Decreased in Tg-B2++*ApoE–/–* Mice

**Figure 4:**
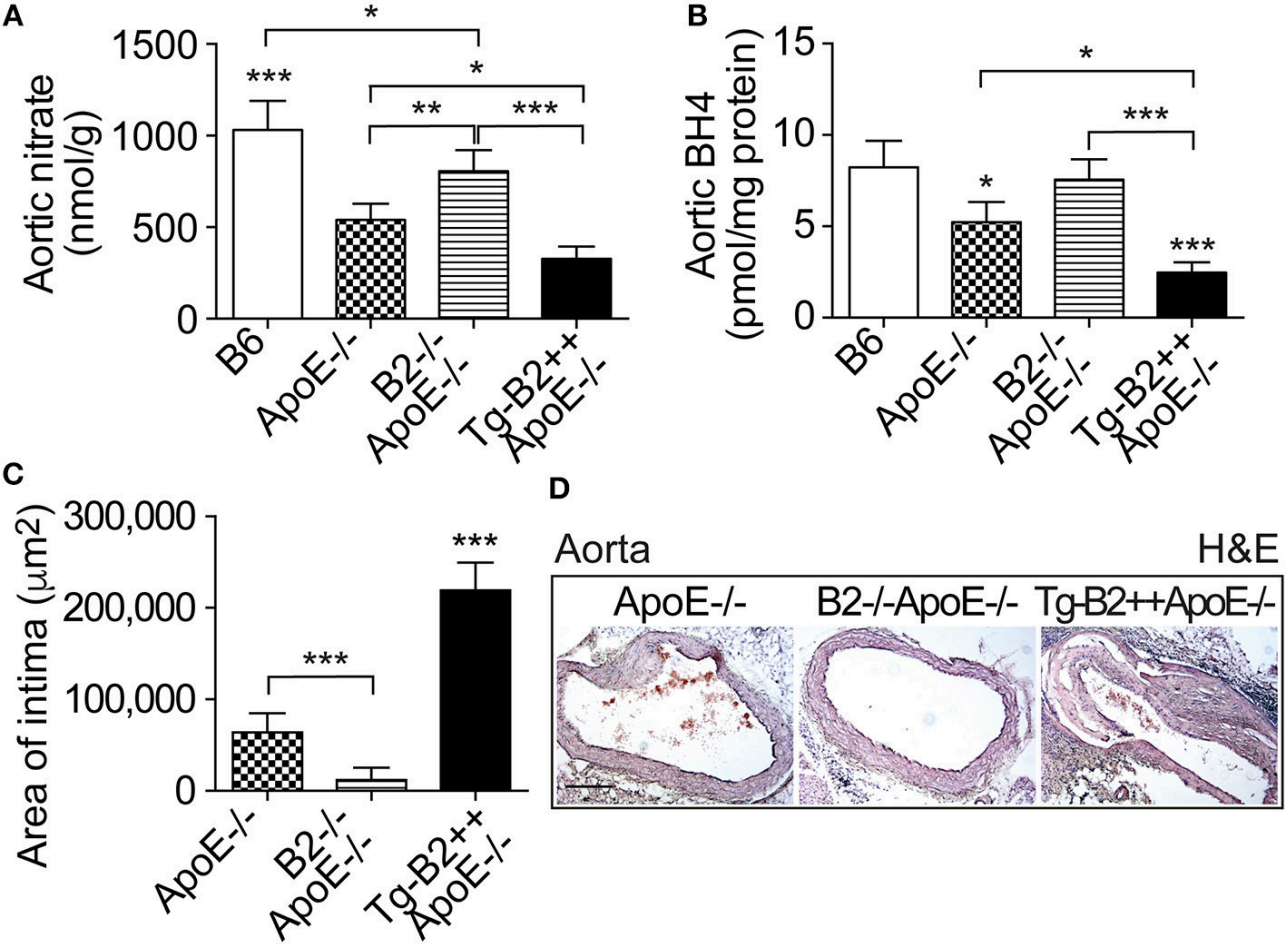
The aortic nitrate content as a marker of NO activity and the nitric oxide synthase (NOS) cofactor tetrahydrobiopterin (BH4) are decreased in Tg-B2++*ApoE–/–* mice. **(A,B)** Aortic nitrate **(A)** and aortic BH4 **(B)** contents of study groups (mean ± s.d.; *n* = 5; ^*^p < 0.05; ^**^p < 0.01; ^***^p < 0.001 vs. *ApoE*–/– and TgB2++*ApoE*–/– **(A)**; mean ± s.d.; *n* = 4; ^*^p < 0.05 vs. B6 and B2–/–*ApoE*–/–, and ^***^p < 0.001 vs. B6 **(B)**; other comparisons are indicated; Tukey‘s test). **(C,D)** Intimal atherosclerotic lesion area was determined on hematoxylin and eosin- (H&E)-stained paraffin sections of the aortic arch. Quantitative data evaluation is shown in **(C)**, and **(D)** shows representative sections (mean ± s.d.; *n* = 3; ^***^p < 0.001 vs. *ApoE*–/– and B2–/–*ApoE*–/–; other comparisons are indicated; Tukey‘s test).

On vascular endothelial cells, the B2 bradykinin receptor is known for its nitric oxide (NO)-stimulating function (10, 28, 29). Nitric oxide is considered as a major atheroprotective factor released from intact endothelium (30, 31). On the other hand, cardiovascular risk factors such as hypercholesterolemia and atherosclerosis trigger endothelial dysfunction (29, 30), which leads to endothelial nitric oxide synthase (eNOS) uncoupling (19). Uncoupled eNOS produces atherogenic superoxide instead of atheroprotective NO (19). In agreement with previous data on defective NO generation in hypercholesterolemic *ApoE*–/– mice (32), the aortic tissue nitrate content as a marker of NO activity *in vivo* (33), was significantly decreased in *ApoE*–/– mice compared to non-transgenic B6 mice, i.e the aortic nitrate of ApoE–/– mice was 539 ± 89 nmol/g compared to 1031 ± 158 nmol/g in B6 mice (Figure 4A). Transgenic expression of *BDKRB2* further decreased the aortic nitrate content in Tg-B2++*ApoE*–/– mice compared to *ApoE*–/–mice with endogenous *Bdkrb2* expression (Figure 4A). Vice versa, deficiency of *Bdkrb2* led to a significantly increased aortic nitrate content of *Bdkrb2*–/–*ApoE*–/– mice compared to *ApoE*–/– mice (Figure 4A). This finding is complementary to previous data, which show that deficiency of *Bdkrb2* increases the serum nitrate level (34). Nevertheless, the aortic nitrate content of *Bdkrb2*–/–*ApoE*–/– mice was not normalized to B6 control level by B2 bradykinin receptor deficiency (Figure 4A), most likely because defective NO generation in atherosclerotic *Bdkrb2*–/–*ApoE*–/– mice has additional causes, which are independent of *Bdkrb2*, e.g., *ApoE* deficiency-induced hypercholesterolemia. Together these data show that the B2 bradykinin receptor mediates a decrease in aortic nitrate content as a marker of dysfunctional aortic (e)NOS activity in Tg-B2++*ApoE*–/– mice whereas deficiency of *Bdkrb2* increases the aortic nitrate level in *Bdkrb2*–/–*ApoE*–/– mice.

The eNOS uncoupling during the pathogenesis of atherosclerosis was partially attributed to down-regulation of the (e)NOS cofactor, tetrahydrobiopterin, BH4 (35, 36). We asked whether the decreased aortic nitrate content of *BDKRB2*-transgenic Tg-B2++*ApoE*–/– mice was accompanied by changes in aortic BH4 levels. We measured the aortic BH4 content and found that transgenic *BDKRB2* expression led to a decrease in the aortic BH4 content of Tg-B2++*ApoE*–/– mice, which was 2.48 ± 0.56 pmol/mg in Tg-B2++*ApoE*–/– mice compared to 5.23 ± 1.09 pmol/mg in *ApoE*–/– mice with endogenous *Bdkrb2* expression level (Figure 4B). For comparison, aortic BH4 levels were not significantly different between *ApoE*–/– mice with *Bdkrb2*-deficiency and non-transgenic B6 mice (Figure 4B). This observation could be due to the fact that there are two opposing mechanisms acting on the aortic BH4 level in *Bdkrb2*–/–A*poE*–/– mice, i.e., (i) *ApoE* deficiency, which decreases the aortic BH4 content, and (ii) *Bdkrb2* deficiency, which counteracts the *ApoE* deficiency-induced BH4 decrease. The net result is a normalization of aortic BH4 content in *Bdkrb2*–/–*ApoE*–/– mice toward the level of non-transgenic B6 mice (Figure 4B).

Histologic evaluation of hematoxylin-eosin-stained aortic specimens from the different groups of *ApoE*–/– mice confirmed the enhanced atherosclerosis progression of Tg-B2++/*ApoE*–/– mice by an increased intimal atherosclerotic lesion area in the aortic arch of Tg-B2++*ApoE*–/– mice with transgenic *BDKRB2* expression compared to *ApoE*–/– mice with endogenous *Bdkrb2* expression, and double-deficient *B2*–/–*ApoE*–/– mice (Figures 4C,D). Together these data show a decrease in (e)NOS activity as documented by a reduced aortic content of nitrate and depletion of the (e)NOS co-factor, BH4, by the B2 bradykinin receptor in hypercholesterolemic Tg-B2++*ApoE*–/– mice with concomitantly enhanced atherosclerotic lesion development.

### The Major BH4-Synthesizing Enzyme, *Gch1* (GTP Cyclohydrolase 1) Is Down-Regulated by *BDKRB2* in Tg-B2++*ApoE*–/– Mice

**Figure 5:**
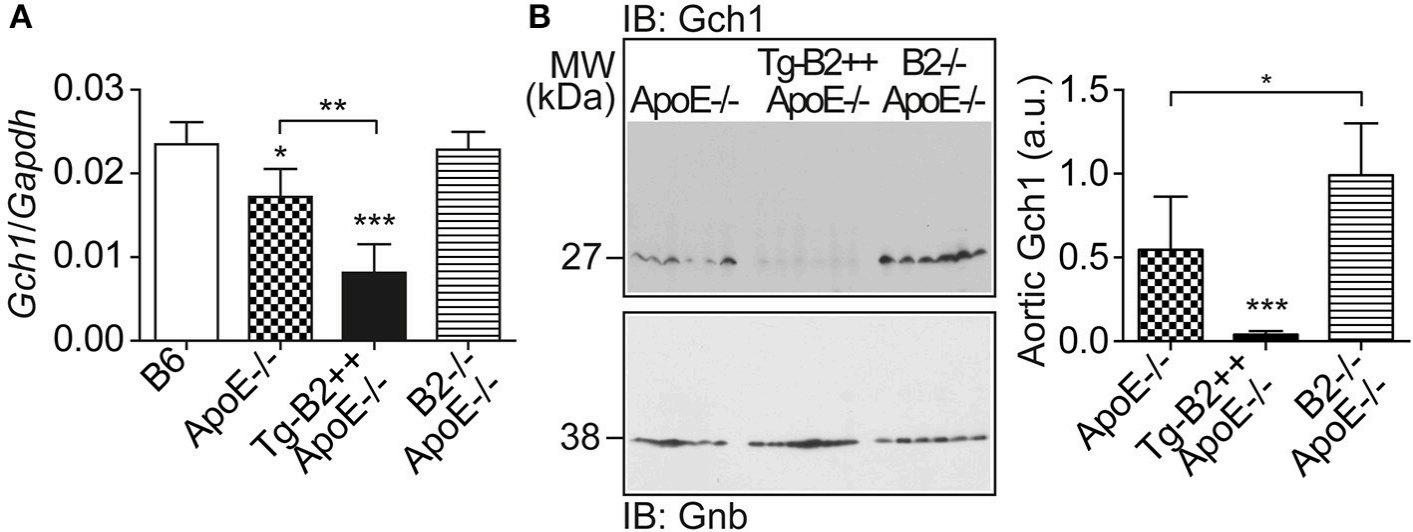
The major BH4-synthesizing enzyme, *Gch1* is down-regulated by *BDKRB2* in Tg-B2++*ApoE*–/– mice. **(A)** Aortic gene expression level of *Gch1* was quantified of indicated study groups (mean ± s.d.; *n* = 4; ^*^p < 0.05 vs. B6; ^**^p < 0.01; ^***^p < 0.001 vs. B6 and B2–/–*ApoE*–/–; other comparisons are indicated; Tukey‘s test). **(B)** The aortic protein content of Gch1 was determined by immunoblot with Gch1-specific antibodies. The left panel shows immunoblot detection of aortic Gch1 of indicated study groups, and the right panel shows quantitative data (mean ± s.d.; *n* = 6; ^*^p < 0.05; ^***^p < 0.001 vs. *ApoE*–/– and B2–/–*ApoE*–/–; other comparisons are indicated; Tukey‘s test).

We searched for the mechanism underlying the aortic decrease in the (e)NOS cofactor, BH4, which was triggered by the B2 bradykinin receptor. BH4 deficiency in atherosclerosis could be caused by at least two different mechanisms, i.e., by (i) BH4 oxidation as a consequence of increased ROS levels (cf. Figure 3), and (ii) impaired BH4 synthesis due to decreased levels/activity of the GTP cyclohydrolase 1 (*Gch1*), which is the rate-limiting enzyme in tetrahydrobiopterin (BH4) biosynthesis (36, 37). Previous data have shown that endothelial *Gch1* activity and expression are decreased by various cardiovascular risk factors (37). Therefore, we determined the aortic expression level of *Gch1* in Tg-B2++*ApoE*–/– mice. Our data show that aortic *Gch1* expression levels were significantly decreased in *BDKRB2*-expressing Tg-B2++*ApoE*–/– mice compared to *ApoE*–/– mice with endogenous *Bdkrb2* levels, i.e., aortic *Gch1* levels were 2.1-fold lower in Tg-B2++*ApoE*–/– mice compared to *ApoE*–/– mice (Figure 5A). For comparison, the aortic *Gch1* expression level of B2–/–*ApoE*–/– mice with *Bdkrb2* deficiency was not significantly different from non-transgenic B6 mice (Figure 5A). As a control, immunoblot detection of the aortic Gch1 protein confirmed the significant decrease of the aortic Gch1 content in *BDKRB2*-expressing Tg-B2++ApoE–/– mice compared to *ApoE–/–* mice with and without endogenously expressed *Bdkrb2* (Figure 5B).

### Treatment With the BH4 Analog, Sapropterin, Retards Atherosclerotic Plaque Formation and Decreases the Aortic ROS Content of Tg-B2++*ApoE*–/– Mice

**Figure 6:**
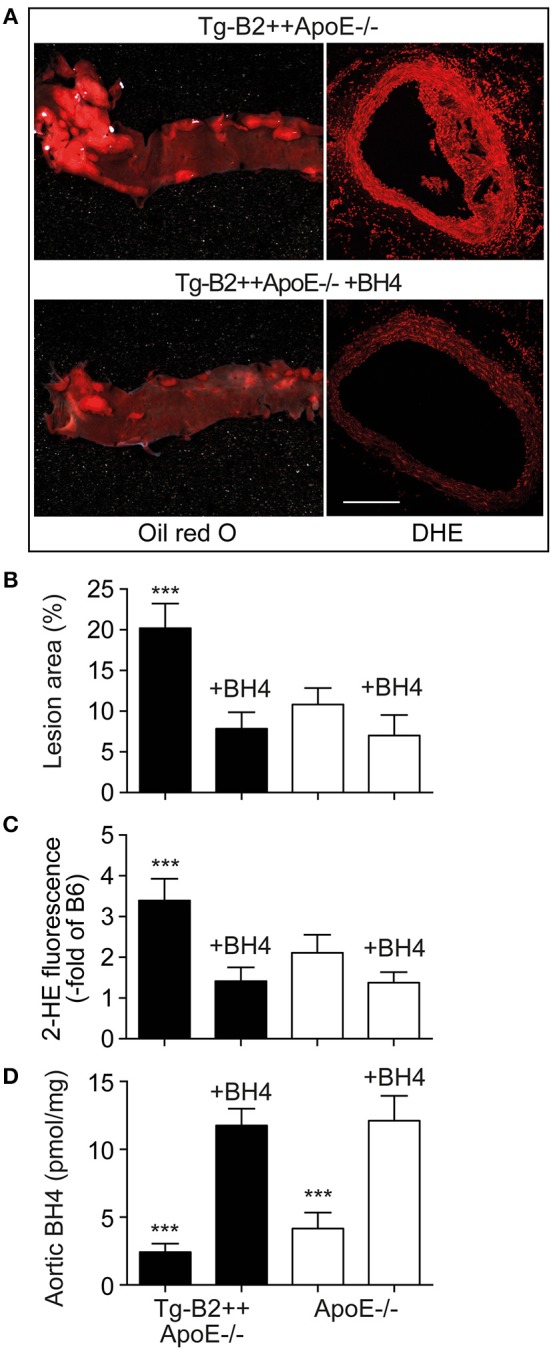
Treatment with the BH4 analog, sapropterin, retards atherosclerotic plaque formation and decreases the aortic ROS content of Tg-B2++*ApoE*–/– mice. **(A–C)** Enhanced atherosclerotic plaque formation of Tg-B2++*ApoE*–/– mice is retarded, and aortic ROS content is decreased by treatment with sapropterin. In **(A)**, representative oil red O-stained aortas (left panels) and DHE-stained aortic sections (right panels) from TgB2++*ApoE*–/– mice are shown (bar: 100 μm; right panels). Panels **(B,C)** present quantitative data (mean ± s.d.; *n* = 4; ^***^p < 0.001 vs. all other groups of mice; Tukey‘s test). **(D)** Aortic BH4 contents of study groups (mean ± s.d.; *n* = 4; ^***^p < 0.001 vs. BH4-treated mice; Tukey‘s test).

Is there a causal relationship between decreased aortic BH4 levels and enhanced atherosclerotic plaque formation in *BDKRB2*-expressing, Tg-B2++*ApoE*–/– mice? To address this question, we treated Tg-B2++*ApoE*–/– mice with the BH4 analog, sapropterin. Treatment outcome of 8-month-old mice was evaluated after 5 months of treatment. Our experiments show that BH4 supplementation retards the enhanced atherosclerotic plaque formation of Tg-B2++*ApoE*–/– mice (Figures 6A,B). In contrast, the effect of BH4 in *ApoE*–/– mice with endogenously expressed *Bdkrb2* was not significant (Figure 6B). Concomitantly, supplementation of BH4 also decreased the exaggerated aortic ROS content of Tg-B2++*ApoE*–/– mice (Figures 6A,C). As a control, saproterin treatment led to increased aortic BH4 levels in Tg-B2++*ApoE*–/– mice and *ApoE*–/– mice with endogenous *Bdkrb2* levels (Figure 6D). These experiments show that BH4 supplementation is capable to counteract the atherosclerosis-promoting ROS generation in Tg-B2++*ApoE*–/– mice with transgenic *BDKRB2* expression.

### ACE Inhibition With Captopril Inhibits Atherosclerotic Plaque Accumulation in Tg-B2++*ApoE–/–* Mice

**Figure 7:**
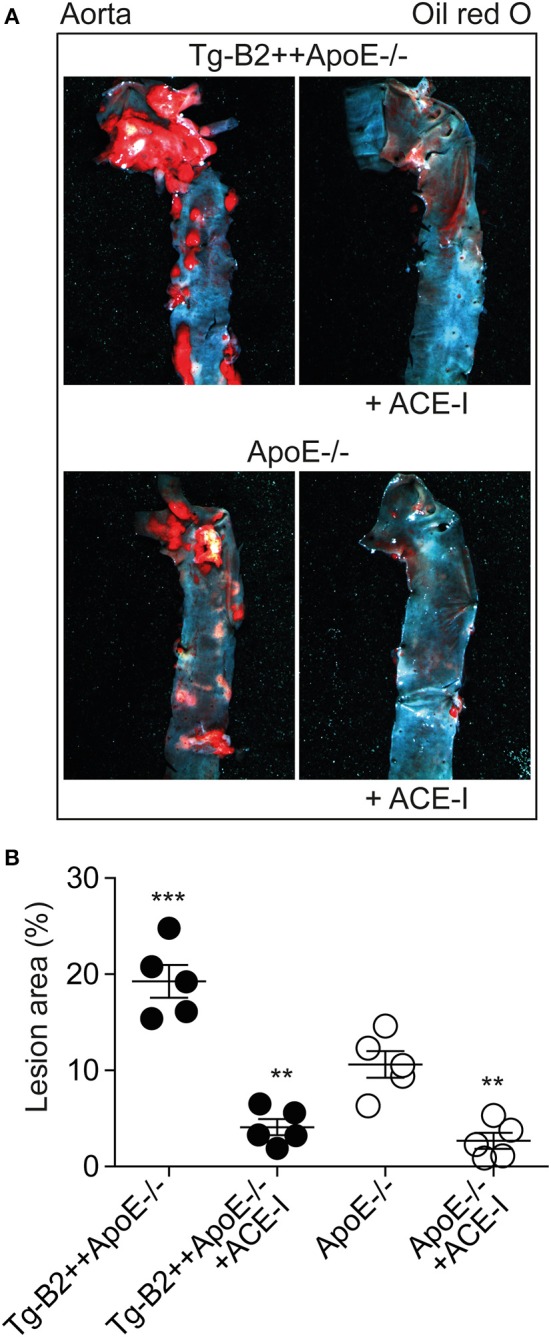
ACE inhibition with captopril inhibits atherosclerotic plaque accumulation in Tg-B2++*ApoE*–/– mice. **(A,B)**. Atherosclerotic plaque area was determined of 8 months-old Tg-B2++*ApoE*–/– and *ApoE*–/– mice after treatment without and with the ACE-inhibitor (+ACE-I), captopril. Panels in **(A)** show representative oil red O-stained aortas, and **(B)** shows quantitative data (mean ± s.d.; *n* = 5; ^**^p < 0.01 vs. *ApoE*–/–; ^***^p < 0.001 vs. Tg-B2++*ApoE*–/–+ACE-I, *ApoE*–/– and *ApoE*–/–+ACE-I; Tukey‘s test).

The angiotensin II AT1 receptor is a major contributor to atherosclerosis-promoting ROS generation in *ApoE*–/– mice (8, 38–40). Atherogenic functions of the AT1 receptor are enhanced by *BDKRB2* (25, 41), and atherosclerosis-related endothelial dysfunction can be prevented by inhibition of angiotensin II AT1 receptor stimulation with an inhibitor of the angiotensin II-generating ACE (42, 43). Because *BDKRB2* enhanced the endothelial dysfunction of Tg-B2++*ApoE*–/– mice, we asked whether inhibition of ACE-dependent angiotensin II generation in Tg-B2++*ApoE*–/– mice could retard the *BDKRB2*-enhanced atherosclerosis progression. To address this question, we treated Tg-B2++*ApoE*–/– mice with the ACE inhibitor, captopril (20 mg/kg/d) for 5 months. Atherosclerotic lesion area was evaluated of oil red O-stained aortas and revealed that captopril largely prevented the accumulation of atherosclerotic plaques in 8 month-old Tg-B2++*ApoE*–/– mice (Figures 7A,B). In agreement with previous studies (7, 8), captopril also inhibited the formation of atherosclerotic lesions in *ApoE*–/– control mice with endogenous *Bdkrb2* expression (Figures 7A,B). Taken together, inhibition of ACE-dependent angiotensin II AT1 receptor activation is capable to prevent the *BDKRB2*-enhanced atherosclerotic lesion development in Tg-B2++*ApoE*–/– mice. Consequently, the atherogenic function of the B2 bradykinin receptor in *ApoE*–/– mice involves a synergistic interplay between the B2 bradykinin receptor and angiotensin II-stimulated AT1 receptor activation.

## Discussion

In this study, we investigated the impact of the B2 bradykinin receptor on atherosclerotic lesion formation in hypercholesterolemic *ApoE*–/– mice as a model of atherosclerosis. We found that a moderately increased *BDKRB2* level in Tg-B2++*ApoE*–/– mice led to a significantly enhanced progression of atherosclerotic lesion development whereas deficiency of *Bdkrb2* retarded the accumulation of atherosclerotic plaques in *Bdkrb2*–/–*ApoE*–/– mice.

In search for pathomechanisms underlying the enhanced atherogenesis triggered by *BDKRB2*, we detected an increased aortic ROS content in Tg-B2++*ApoE*–/– mice compared to *ApoE*–/– mice with endogenous *Bdkrb2* expression level. In addition, the endogenous *Bdkrb2* contributes to enhanced ROS generation in *ApoE*–/– mice because the aortic ROS content was significantly lower in double-deficient *Bdkrb2*–/–*ApoE*–/– mice compared to *ApoE*–/– mice with intact endogenous *Bdkrb2* gene. The increased vascular ROS level could directly contribute to enhanced atherosclerotic lesion formation because ROS is known to accelerate atherogenesis in animal models of atherosclerosis and patients with atherosclerotic vascular disease (8, 18, 27). Aortic ROS generation in *ApoE*–/– mice was previously attributed to the atherosclerosis-enhancing function of the angiotensin II AT1 receptor, which generates ROS by activation of NADPH oxidases (8, 38). Activation of the AT1 receptor in experimental models of atherosclerosis and patients with cardiovascular disease is increased due to hypercholesterolemia-induced up-regulation of the systemic renin angiotensin system (39, 40). Our study identifies the B2 bradykinin receptor as another player involved in enhanced ROS generation in *ApoE*–/– mice. B2 bradykinin receptor-enhanced ROS generation could as well be mediated by the AT1 receptor, which becomes hyperactive by protein complex formation with the B2 bradykinin receptor (25, 41). Notably, endogenous *Bdkrb2* levels are sufficient to sensitize the AT1 receptor-stimulated response, and the AT1-sensitizing function of the B2 bradykinin receptor does not require bradykinin (25, 41). In agreement with a bradykinin-independent function of the B2 bradykinin receptor in atherosclerosis, treatment of *ApoE*–/– mice with the B2 bradykinin receptor-specific antagonist, HOE140, did not alter atherosclerosis progression (13). However, the (brady)kinin-generating kallikrein system was not active in this study, because treatment with the B2-specific antagonist, HOE140, had no effect on blood pressure, neither under basal conditions nor upon blood pressure lowering with the ACE inhibitor, ramipril (13). Thus, bradykinin-dependent and bradykinin-independent effects need to be considered in the atherosclerosis-promoting activity of the B2 bradykinin receptor depending on the activation state of the kinin-kallikrein system.

**Figure 8:**
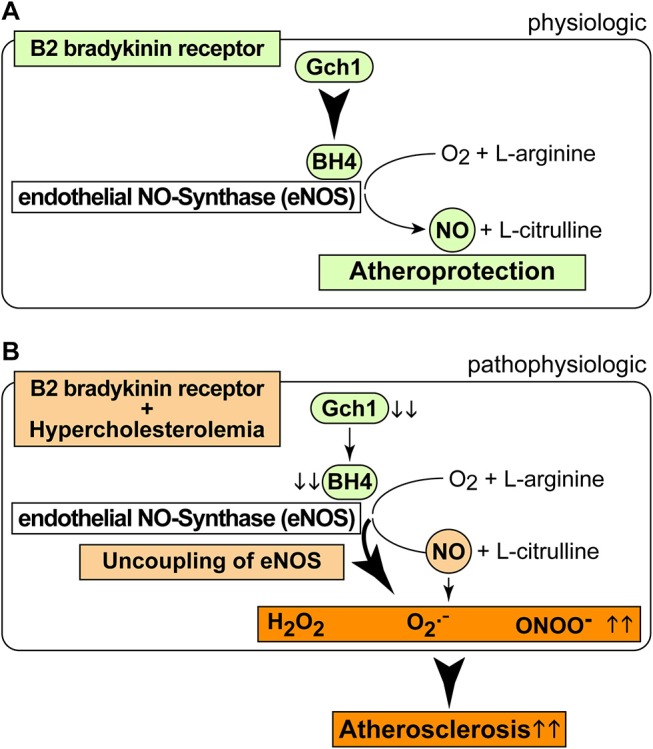
Scheme of physiologic and pathophysiologic functions of the B2 bradykinin receptor. **(A,B)** Physiologic functions of the B2 bradykinin receptor in endothelial cells are atheroprotective **(A)**, and pathophysiologic functions **(B)** of the B2 bradykinin receptor in hypercholesterolemia enhance atherogenesis.

As a consequence of the AT1-receptor sensitizing activity, the B2 bradykinin receptor could mediate a decrease in the nitric oxide synthase (NOS) cofactor, BH4, which is inactivated by ROS (37). Decreased BH4 leads to uncoupled eNOS, which in turn generates more ROS. The decrease in aortic BH4 content could synergistically be aggravated by downregulation of *Gch1*, which is the rate-limiting enzyme in BH4 synthesis. In concert with hypercholesterolemia, *BDKRB2* and *Bdkrb2* could decrease vascular *Gch1* expression and protein level by activation of Gi-coupled signaling because hypercholesterolemia and Gi-coupled signaling are both known to down-regulate *Gch1* (44–46). The ensuing depletion of vascular BH4 could contribute to eNOS uncoupling in the pathogenesis of atherosclerosis (35). The uncoupled eNOS generates detrimental ROS instead of atheroprotective NO (Figure 8). Transgenic animal models support that *Gch1* deficiency and reduced vascular BH4 accelerate atherosclerosis whereas supplementation of BH4 or *Gch1* expression reverse these deficits and retard atherosclerosis (19, 35, 47). In agreement with these findings, we found that treatment with BH4 decreased aortic ROS levels and dampened the atherosclerosis-enhancing effect of *BDKRB2*.

It is well-established that the bradykinin-potentiating effect of ACE inhibitors contributes to cardio-protection [Figure 8A, (9, 10)]. However, the atherosclerosis-lowering potential of ACE inhibition does not necessarily rely on bradykinin-stimulated *Bdkrb2* activation, e.g., when there is no activation of the (brady)kinin-generating kallikrein system (13). A B2-specific antagonist, HOE140, also had no effect on atherosclerosis progression in *ApoE*–/– mice without ACE inhibition (13), most likely because the beneficial NO-generating capacity of endothelial B2 bradykinin receptor stimulation is impaired in atherosclerosis and cardiovascular disease [Figure 8B, (42, 43, 48)]. Because atherosclerosis progression in *ApoE–/–* mice is not affected by the B2-specific antagonist, HOE140 (13), atherosclerosis promotion by *Bdkrb2* in *ApoE–/–* mice could largely be mediated by the angiotensin II AT1 receptor-sensitizing function of *Bdkrb2*, which is bradykinin-independent (25, 41). In agreement with this conclusion, our study shows that inhibition of angiotensin II AT1 receptor stimulation by an ACE inhibitor completely prevents atherosclerotic lesion formation in Tg-B2++*ApoE*–/– mice. Thus, under hypercholesterolemia, the B2 bradykinin receptor gene is atherogenic, leads to increased vascular ROS, mediates a decrease in (e)NOS activity as evidenced by a reduced aortic nitrate content, and enhances aortic BH4 deficiency (Figure 8B). BH4 depletion contributes to the atherogenic activity of the B2 bradykinin receptor because supplementation with BH4 was sufficient to inhibit aortic ROS and retard atherosclerosis progression triggered by transgenic *BDKRB2* expression (Figure 8B).

Enhanced ROS generation in hypercholesterolemia and atherosclerosis is largely attributed to angiotensin II AT1 receptor stimulation (8, 38). Consequently, inhibition of detrimental AT1 receptor-stimulated ROS generation by an ACE inhibitor or AT1 antagonist in animal models and patients could exert a dual protective function regarding the atherogenic activity of *BDKRB2*, i.e., (i) restoration of protective B2 bradykinin receptor signaling, and (ii) neutralization of the AT1-sensitizing function of *BDKRB2*. Symptoms of the herein deduced atherogenic activity of the B2 bradykinin receptor can be prevented by ACE inhibition, which inhibits sensitized AT1 receptor signaling, blunts exaggerated ROS generation, and heals uncoupled eNOS in atherosclerosis and cardiovascular disease (42, 43, 47). Consequently, the agonist-stimulated B2 bradykinin receptor could become anti-atherogenic upon treatment with an ACE inhibitor, which prevents endothelial dysfunction triggered by oxidized low-density lipoproteins in a B2 bradykinin receptor stimulation-dependent manner (49).

Taken together, our study shows that the atherosclerosis-enhancing function of the ubiquitously expressed B2 bradykinin receptor involves a synergistic interplay between enhanced ROS generation, dysfunctional NO activity and AT1 receptor activation. Pathomechanisms identified in this study could act in concert with already established B2 and/or AT1 receptor-dependent atherogenic activities. Notably, the following mechanisms mediated by the B2 and/or AT1 receptor could play a direct or indirect role in B2 bradykinin receptor-enhanced atherosclerosis: (I) The B2 bradykinin receptor could promote atherosclerosis by the reduced activation of atheroprotective AT1-inhibitory receptors, *AGTR2* and *MAS1* (34, 50–52), which are directly down-regulated by the B2 bradykinin receptor and angiotensin II AT1 receptor (34, 53) and indirectly dampened via angiotensin II AT1-mediated *ACE2* down-regulation (54). (II) In addition, the B2 bradykinin receptor could enhance atherogenesis by decreasing the expression of the vasculoprotective endothelial Kruppel-like factor 4, *KLF4* (55), which is directly downregulated by angiotensin II AT1 stimulation [(56); NCBI GEO dataset GSE19286 in Abd Alla et al. (7)], and indirectly down-regulated by reduced Mas receptor activation (57). (III) And finally, the B2 bradykinin receptor-stimulated atherogenesis could involve the enhanced aortic infiltration with macrophages and inflammatory immune cells, which is promoted by angiotensin II AT1 receptor signaling directly (7, 58), and indirectly by B2 bradykinin and angiotensin II AT1 receptor-mediated downregulation of Mas, which accounts for a decreased expression of atheroprotective sirtuin 1, *SIRT1* (34, 57, 59). In view of this panoply of different atherogenic functions, specific targeting of the detrimental B2 - AT1 receptor axis could be envisaged as a potential approach to treat not only atherosclerosis but also related pro-thrombotic activities triggered by the B2 bradykinin and angiotensin II AT1 receptors (34, 60, 61).

## Author Contributions

AP, SW, YJ, AL, and JA performed experiments. JA generated transgenic mice. All authors evaluated data. UQ conducted the study, designed experiments and wrote the manuscript. All authors read and approved the final version of the manuscript.

### Conflict of Interest Statement

The authors declare that the research was conducted in the absence of any commercial or financial relationships that could be construed as a potential conflict of interest. The handling editor and reviewer JBP declared their involvement as co-editors in the Research Topic, and confirm the absence of any other collaboration.
